# Scopus based bibliometric and scientometric analysis of occupational therapy publications from 2001 to 2020

**DOI:** 10.12688/f1000research.108772.1

**Published:** 2022-02-08

**Authors:** Koushik Sau, Yogendra Nayak

**Affiliations:** 1 Department of Occupational Therapy, Manipal College of Health Professions, Manipal Academy of Higher Education, Manipal, Karnataka, 576104, India; 2 Department of Pharmacology, Manipal College of Pharmaceutical Sciences, Manipal Academy of Higher Education, Manipal, Karnataka, 576104, India

**Keywords:** Occupational therapy, occupational therapy journals, core journals, citations metrics, Scopus, VOSviewer

## Abstract

**Background:** Occupational therapy (OT) is one of the allied health professions that had its first journals way back in 1920. The main objective of this study was to find out the publication trend in the field of OT research for the period of 2001-2020 using the principles of bibliometrics and scientometrics.

**Methods:** The data was retrieved from Scopus from the past 20-years (2001-2020). VOSviewer software was used to find year-wise publications in OT-specific and non-OT-specific Journals along with top journals, countries, organisations, authors, cited articles, and highly used keywords.

**Results:** There was a steady growth of OT articles from the past 20-years. Scopus indexes 16 OT-specific journals are identified. American Journal of Occupational Therapy, British Journal of Occupational Therapy Journal, Australian Occupational Therapy Journal, Scandinavian Journal of Occupational Therapy and Canadian Journal of Occupational Therapy are the leading publications and citations. Comparison of OT-specific and non-OT journals inferred that the OT-specific papers are three times more published in non-OT journals. There is a trend in publishing multidisciplinary medical journals than OT journals. The US publishes the highest number of articles, followed by the UK, Australia, Canada, and Germany. Though the US alone produced a considerable number of articles (9517), only five organisations are listed in the top-20, compared to Canada (n=6) and Australia (n=5). Australia represents the highest published authors (n=11/20), and Canada represents a highly cited author from the top-cited publications. The “occupational therapy”, “rehabilitation”, “stroke”, “physical therapy,” and “activities of daily living” are the five common keywords used by OT authors. This study lists top-20 journals along with their CiteScore and Journal Impact Factor.

**Conclusions:** This study will help the budding researchers in OT to select a suitable quality journal for publication and, further, helpful for research promotion, researcher incentivising, grant allocations, and policymakers in the OT field.

## Introduction

Occupational therapy (OT) is one of the allied health professions practiced globally. Research and research publications are an integral part of the OT profession. *“Archives of Occupational Therapy”* was the first published OT journal published in 1922 ( Cruz *et al.*, 2019). In the 21 ^st^ century, most of the journals became online web-based after the invention of the internet ( Cruz *et al.,* 2019). There are thirty-nine journals published that have contained “occupational therapy” in journal-title available online. Thirty-two among those journals are published in the English language ( Cruz *et al.,* 2019). These journals are core OT journal that mainly publishes OT-related articles. With the advancement in electronic databases and the online availability of periodicals, the newer bibliometrics and scientometrics methods of journal evaluation or evaluation of research measures are evolved ( Meho and Yang, 2006). Citation analysis and content analysis are two commonly used methods of bibliometric analysis ( Wallin 2005; T. Brown, Gutman, Ho, *et al.*, 2018). This method is proven to examine the impact or influence of published articles, journals, researchers, institutions, and countries. Further, this method evaluates the performance in micro-level such as institution/university performance to macro-level such performance of a particular profession in research and country-wise research evaluation ( Wallin 2005; T. Brown, Gutman, Ho, *et al.* 2018).

Content and citation analysis is a common practice in bibliometrics of occupational therapy. In the past, most of the content analysis was performed for specific OT journals such as the *American Journal of Occupational Therapy* (AJOT) ( Ottenbacher and Short, 1982; Ottenbacher and Petersen, 1985), *Canadian Journal of Occupational Therapy* (CJOT) ( Ernest, 1983), *Australian Occupational Therapy Journal* (AOTJ) ( Trevan-Hawke, 1986; Madill *et al.*, 1989), *British Journal of Occupational Therapy Journal* (BJOT) ( Cusick, 1995; Mountain 1997) and *Occupational Therapy Journal of Research* (OTJR) ( G. T. Brown and Brown, 2005). Pearl *et al.* (2014) evaluated and reported the content of five occupational therapy journals as AJOT, AOTJ, BJOT, CJOT, and *Scandinavian Journal of Occupational Therap*y (SJOT).

Most of the reported studies were based on the cited journal analysis, that is, analysis of what OT journals articles cited from other journals ( Johnson and Leising, 1986; Roberts, 1992; Reed, 1999; Potter, 2010). Recently, Nowrouzi-Kia *et al.,* (2018) evaluated top publications with more than 100-citations in occupational therapy. This study was the first to uncover the highest annual citation rates, randomised control trials, literature reviews, and cross-sectional studies in occupational therapy.

Ziviani and colleagues (1984) analysed the content and citation of three OT journals, *AJOT, BOTJ,* and *AOTJ.* It was the first bibliometric study examining both content and authority in the OT field. In the recent past, bibliometric studies have become a popular method to know the publication trends, find researchers/authors in a particular topic, and identify research journals where prospective authors select to publish their manuscripts. This type of study helps identify the publication landscape of a specific profession ( MacDermid *et al.*, 2015). These data are also used as criteria for research promotion, researcher incentivizing, grant allocations, and policymaking. Further, these data can also be used to benchmark faculty, department, and institute or research organization ( T. Brown *et al.*, 2019).

Several studies were reported on the performance of OT researchers. Those studies evaluate OT authors' publication output in terms of their content and citations impact. All analyses were conducted for OT authors from specific geographical locations such as Canada ( MacDermid *et al.*, 2015), Brazil ( Folha *et al.*, 2017), the United Kingdom ( T. Brown, Ho *et al.*, 2018), and Australia ( T. Brown, Gutman, and Ho, 2018). Recently one study reported the bibliometric analysis for authors of western countries and Asian countries. It includes a total of nine countries among them, United States (US), Canada, the United Kingdom (UK), and Australia were included as countries of the west, and Hong Kong, Japan, Taiwan, Singapore, and South Korea were included as Asian countries ( Man e *t al.*, 2019). Among those few publications, it was observed that OT authors had published a high number of publications in non-OT journals ( MacDermid *et al.*, 2015; T. Brown *et al.*, 2017; T. Brown, Ho, *et al.*, 2018; T. Brown, Gutman, and Ho, 2018). A good percentage of highly cited OT papers, also published in non-OT-specific journals ( Man *et al.*, 2019). Another study on OT-publication in non-OT journals for 2004 to 2015 found publication in non-OT journals increased by 173% ( Folha *et al.*, 2019). Because there has been an explosion of new online electronic journals with open access options for publications, publishing is a multidisciplinary type of journal that has become the global trend by prospective authors in any discipline.

Recently all bibliometric studies in the field of OT used Journal Citation Report (JCR) matrices and Journal Impact Factor (JIF) for their analysis provided by Web of Science (WOS) ( T. Brown, Gutman, Ho, *et al.*, 2018; MacDermid *et al.*, 2015; Gutman *et al.*, 2017; Augusto De Araujo *et al.*, 2017; T. Brown *et al.*, 2017; T. Brown *et al.*, 2019; T. Brown, Gutman, and Ho, 2018; Man *et al.*, 2019). JIF is calculated by a simple mathematical formula, where the total citations will be in the numerator and the citable item as the denominator for the previous two years. The denominator creates confusion because the authors cite anything from the available literature, and journals do not label the publications as citable items in their author guidance. Neither prevents any kind of item for citing ( Fernandez-Llimos, 2018). Similar to JIF, Scopus CiteScore also use citation analysis for ranking journals. The Scopus database that provides CiteScore is among the two databases accepted worldwide. The other is Web of Science (WOS), which provides JIF ( T. Brown and Gutman, 2019; Fernandez-Llimos 2018; Sau, 2020).

The CiteScore metrics released in 2017 offer access to citing and citable articles, and it computes all published materials as citable articles ( Fernandez-Llimos, 2018). But, there was concern about citable items such as “notes”, “letter to the editor”, “editorials”, “erratum”, “retracted”, and there will be many unidentified items. This error has been fixed in the recently updated version of CiteScore metrics 2020, where they have fixed for the “articles”, “reviews”, “conference papers”, “book chapters” and “data papers” published. Scopus CiteScore has more excellent comprehensive coverage of published research data ( Fernandez-Llimos, 2018; Sau, 2020). Both JIF and CiteScore calculations are based on citations received by a journal in a given period for the published citable item in that duration ( Fernandez-Llimos, 2018; Sau, 2020). Significant and strong correlations were found between JIF and CiteScore in recent studies comparing 14-OT journals published in English ( T. Brown and Gutman, 2019). Hence, both JIF and CiteScore for measuring journal quality are used in our current study.

Thus, it indicates a requirement for a comprehensive bibliometric evaluation of OT literature using accurate and accessible matrices, which can be used to understand the global prospect of OT literature. These reasons prompted us to conduct a detailed bibliometric and scientometric analysis of research output in OT using the Scopus database. Hence, the following research questions were proposed.

i) What is the year-wise OT-publications trend in Scopus indexed journals for 2001-2020?
ii) What are the trend in OT-specific journals and non-OT-specific journal publications for 2001-2020?
iii) Which are the top journals that publish peer-reviewed OT publications between 2001 and 2020?
iv) Which are top-performing countries in OT-research publications between 2001 and 2020?
v) What are the collaboration trends in OT for 2001-2020?
vi) Which are the top-performing organisations in OT for the period of 2001-2020?
vii) Which are the top-cited publications in OT for 2001-2020?
viii) Which are the commonly used keywords in OT journals for 2001-2020?

## Methodology

### Ethical approval

This study was conducted based on data retrieved from Scopus between 2001 and 2020. Due to no human subjects involvement, ethical approval was not required.

### Source of data

Scopus database was used for the study. Scopus is one of the international scientific committee's best bibliographic databases ( Fernandez-Llimos, 2018; Sau, 2020). Scopus covers 70 million items and 1.4 billion cited references since 1970, making it the biggest research publication database. Scopus bibliometric information is expensively used in scientometric analysis.

### Data retrieval strategies

The search was conducted in December 2021 and was limited to scientific articles and reviews on OT published from 2001 to 2020. We used the TITLE-ABS-KEY function using the advance search option of the Scopus database. Keywords such as *“Occupational Therapy”* and *“Occupational Therapist”* ( T. Brown, Gutman, Ho, *et al*., 2018; Nowrouzi-Kia *et al.*, 2018; T. Brown *et al.*, 2019; T. Brown *et al.*, 2017: Gutman *et al.*, 2017) was used along with multiple Boolean operators such as “AND”, and “OR” to retrieve the maximum number of relevant articles. We excluded non-peer-reviewed documents such as “short survey”, “conference paper”, “editorial”, “notes”, “letter”, “book”, “book chapter”, “erratum” and “retracted papers” ( T. Brown, Gutman, Ho, *et al.*, 2018; Nowrouzi-Kia *et al.*, 2018).

### Scientometric analysis

The VOSviewer®, a free software, was used. Microsoft Excel 2016® was used for all the figures and scientometric calculations.

Scientometric data was retrieved from Scopus using a comma-separated file (CSV) with complete information. The full details such as citation information, bibliographical information, abstract and keywords, funding details, and other pieces of information were downloaded as CSV-file to analyse the data using VOSviewer.

### Identification of year-wise publication trends

All journal titles were screened to identify the OT-specific journals from the Scopus database. The journals with titles that contained the “Occupational therapy” word were categorised as OT-specific journals ( Folha *et al.*, 2019). Additionally, we verified all journals' title, which contains either of these three words “Ergotherapia”, “Occupational Therapy”, or “OT” and confirmed the organisation that published those journals. Further, the occupational therapy organisation or association publishing journals were included in the OT-specific journal list. The remaining journals were categorised as non-OT-specific journals. Then year-wise CSV file was imported into VOSviewer to generate year-wise publication of OT-specific, non-OT specific information. We used VOSviewer software citation per source function for this purpose. VOSviewer uses “source” instead of “journal” in software. Citation per publication is used to generate ten-year journal publication output. Then the information is imported to an Excel file to compute year-wise OT publications, year-wise publications in OT-specific journals, and year-wise publications in non-OT specific journals. The cumulative publications for all three categories were calculated separately to identify the growth trend of OT publications.

### Identifications of top journals, authors, countries, and organisations

The VOSviewer was used to categorise the top twenty journal sources, countries, and 20 organisations based on publication numbers. Similarly, we used twenty-year files to identify the top twenty authors who published a minimum of 25 articles and the top 20 individual articles based on the number of citations. We referred to analysis using CiteScore 2020 from the Scopus database and Journal Citation Report 2020 from WoS Master Journal List respectively to tabulate the Scopus CiteScore and JIF for the top-twenty journals.

### Country collaboration network and authors keyword visualisation

A network map on international collaboration about OT publication and author keywords visualisation was generated using VOSviewer software.

## Results

**Figure 1.**
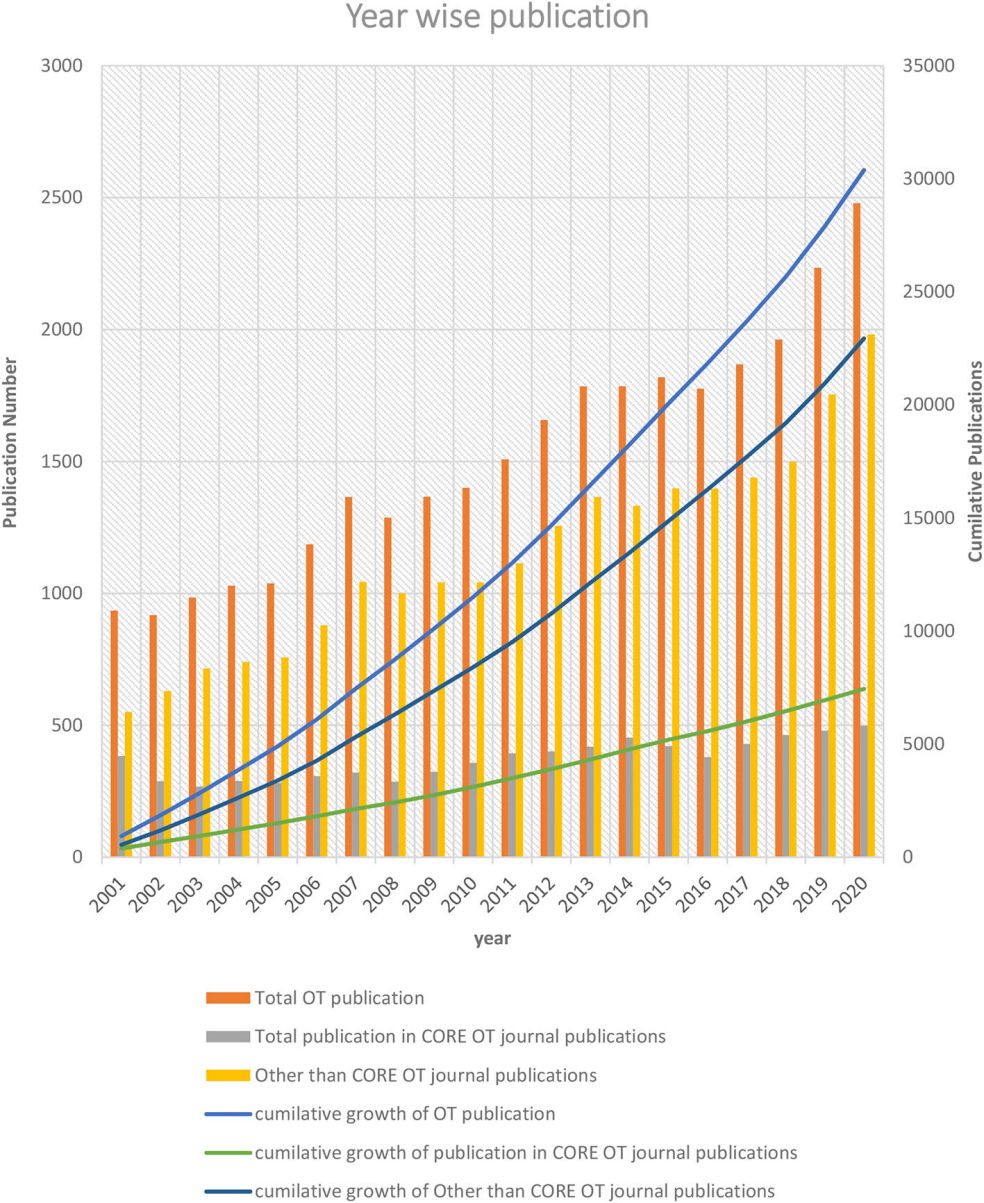
Year-wise OT-publication trend.

The overall OT publication trends revealed a steady growth of (7.72%) each year. In OT-specific and non-OT specific journals publication, growth was 0.29% and 7.44%, respectively ( Figure 1). Approximately one-fourth (24.30%) of articles were published in OT-specific journals in the last twenty years, and three-fourths (75.69 %) articles were published in non-OT-specific journals. From 2001 to 2020 the OT publications increased by 165.14 % (n = 1544). Similarly, the overall OT publication in OT-specific journals increased 14.84 % (n=57), whereas non-OT-specific journals overall publication increased 269.85% (n =1487).

**Figure 2.**
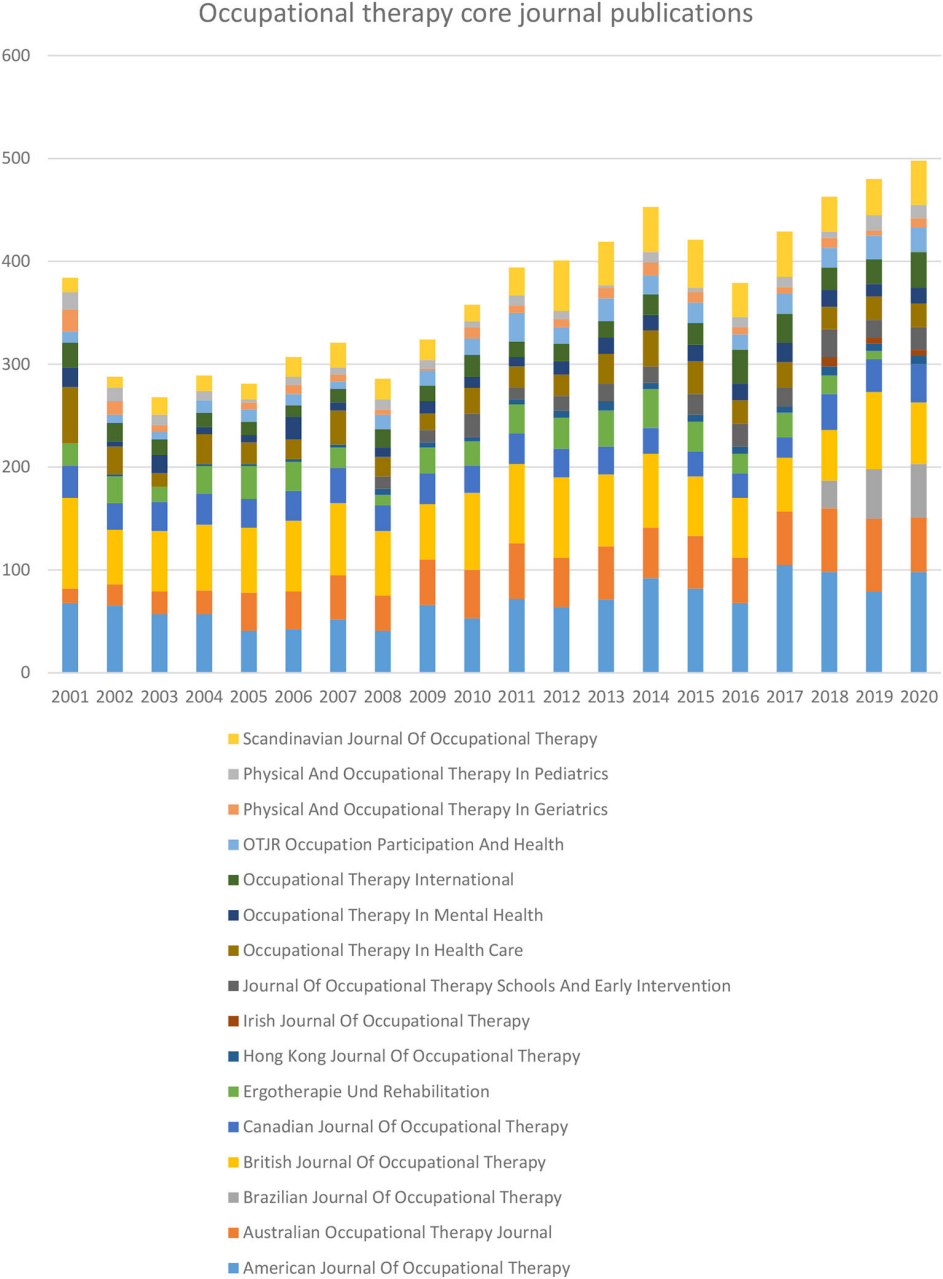
Year-wise occupational therapy specific journal publication.

A total of 16 OT-specific journals are listed in the Scopus database. Year-wise OT specific journal publication for the period of 2001 to 2020 showed in Figure 2. The top five journals published more than half of OT-specific journal publications. Those journals were American *Journal of Occupational Therapy* (AJOT) 18.42 %, *British Journal of Occupational Therapy* (BJOT) 17.56 %, *Australian Occupational Therapy Journal* (AOTJ) 11.53%, *Canadian Journal of Occupational Therapy* (CJOT) 7.64 % and Scandinavian *Journal of Occupational Therapy* (SJOT) 7.64 %, ( Table 1). Reaming articles were published *in Occupational Therapy in Health Care* (OTHC) 6.87 (n=511), *Ergotherapie und Rehabilitation* (ErgoR) 6.15 % (n=458), *Occupational Therapy International* (OTI) 5.28 %(n=393), *OTJR Occupation, Participation and Health* (OTJR) 4.27 % (n=318), *Occupational Therapy in Mental Health* (OTMH) 3.56% (n=265), *Journal of Occupational Therapy, Schools, and Early Intervention* (JOTSEI) 3.10 % (n=231), *Physical and Occupational Therapy in Pediatrics* (POTP) 2.42% (n=180), *Physical and Occupational Therapy in Geriatrics* (POTG) 2.24% (n = 167), *Brazilian Journal of Occupational Therapy* (BrJOT) 1.71 % (n=127), *Hong Kong Journal of Occupational Therapy* (HKJOT)1.32% (n =98), and *Irish Journal of Occupational Therapy* (IrJOT) 0.28 % (n=21).

**Table 1.** Top-20 occupational therapy specific journals.

Sl no	Source	ISSN	Journal Impact Factor (JIF) 2020/Journal Citation Indicator (JCI)	Cite score 2020	Documents	citations	cpd
1	British Journal of Occupational Therapy	0308-0226	1.243	1.8	1307	13489	10.32
2	American Journal of Occupational Therapy	0272-9490	2.246	2.6	1259	27770	22.06
3	Australian Occupational Therapy Journal	0045-0766	1.856	2.1	857	12200	14.24
4	Scandinavian Journal of Occupational Therapy	1103-8128	2.611	2.7	568	7916	13.94
5	Canadian Journal of Occupational Therapy	0008-4174	1.614	1.9	560	10519	18.78
6	Occupational Therapy in Health Care	0738-0577	0.53	1.3	511	3606	7.06
7	Ergotherapie Und Rehabilitation	0942-8623	NA	0	458	93	0.20
8	Occupational Therapy International	0966-7903	1.448	1.5	392	5662	14.44
9	Work	1051-9815	1.505	1.8	340	3149	9.26
10	Disability and Rehabilitation	0963-8288	3.033	3.9	331	6566	19.84
11	OTJR Occupation, Participation and Health	1539-4492	1.768	2.2	304	3775	12.42
12	Occupational Therapy In Mental Health	0164-212X	0.32	1.5	264	1601	6.06
13	Archives of Physical Medicine and Rehabilitation	0003-9993	3.966	5.7	256	9531	37.23
14	Journal of Occupational Therapy, Schools, and Early Intervention	1941-1243	0.22	0.8	231	807	3.49
15	Journal Of Allied Health	0090-7421	NA	0.9	179	2019	11.28
16	International Journal of Therapy and Rehabilitation	1741-1645	0.18	0.5	178	782	4.39
17	Physical and Occupational Therapy in Pediatrics	0194-2638	2.36	2.9	178	3615	20.31
18	Physical and Occupational Therapy In Geriatrics	0270-3181	0.26	0.7	169	1017	6.02
19	Clinical Rehabilitation	0269-2155	3.477	4.9	157	5671	36.12
20	Brazilian Journal of Occupational Therapy	2526-8910	0.13	0.4	127	168	1.32

The current analysis found that 14 out of the top-20 journals are OT-specific journals, which published one-fourth of OT articles from 2001 to 2020 ( Table 1). Among those journals, the AJOT received more citations (n = 27770), but the *Archives of Physical Medicine and Rehabilitation* (APMR) received the highest citation per document (37.27). CiteScore of those 20 journals ranges from minimum zero to a maximum of 5.7. Fifteen journals out of the top-20 journals have journal impact factors (JIF) or journal citation indicators (JCI). Six journals were indexed in the Emerging Sources Citation Index (ESCI) of WOS, whereas two journals were not listed in WOS. Impact factors for those 15 journals were rage from 0.03 to 3.966.

**Figure 3.**
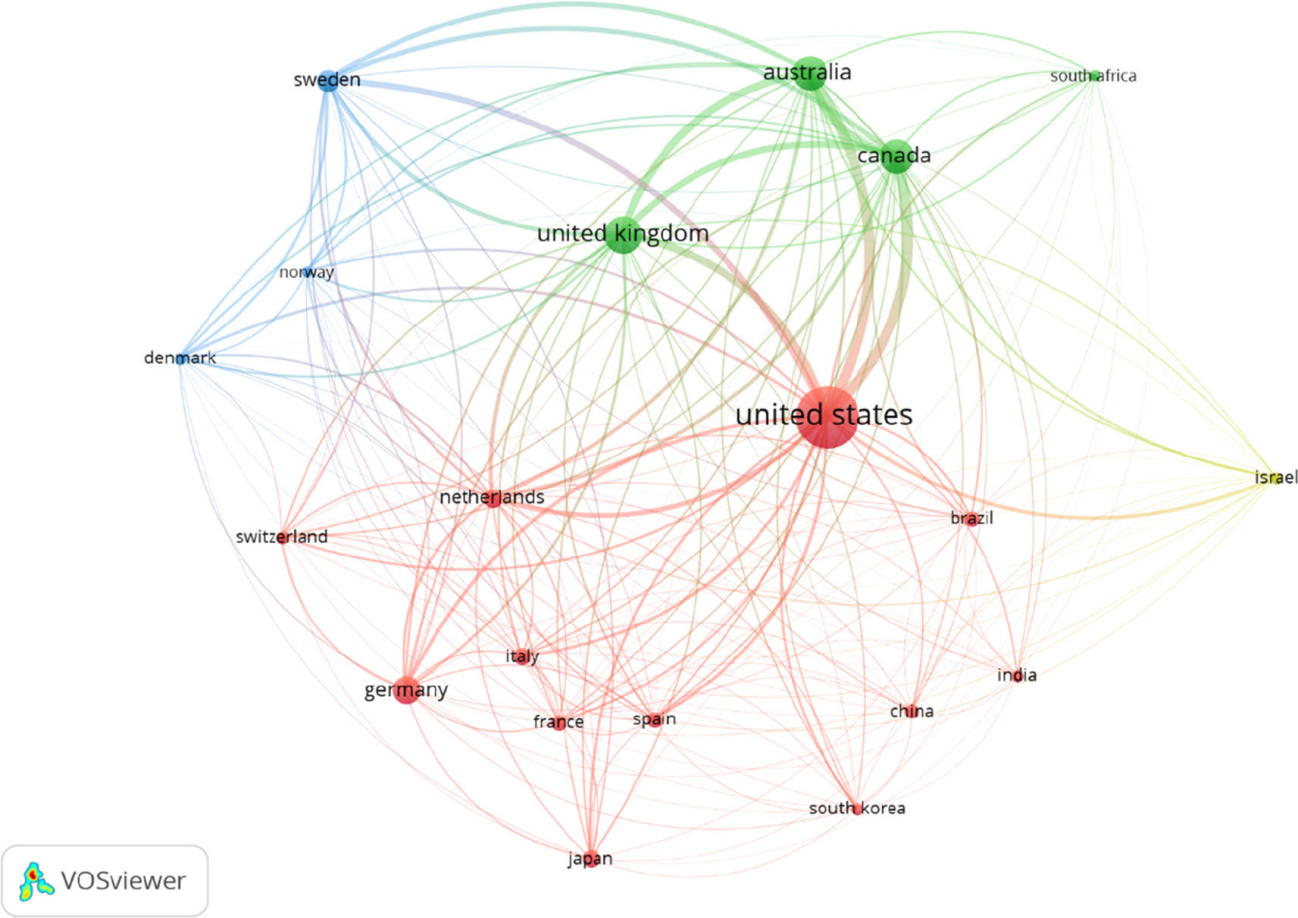
Occupational therapy publication collaboration-network of top-20 publishing countries.

Among the top OT publishing countries, the US published the highest number of articles (n= 9517), followed by the UK, Australia, Canada, and Germany ( Table 2). The Netherlands received the highest citation per document (41.84), followed by Denmark, France, Italy, Norway. Furthermore, data revealed the US, UK, Australia, and Australia have solid international collaboration among the best 20 countries published in the past 20 years ( Figure 3).

**Table 2.** Top-20 countries in occupational therapy publications.

Sl	country	documents	citations	cpd
1	United States	9517	216810	22.78
2	United Kingdom	3500	86130	24.61
3	Australia	2887	62476	21.64
4	Canada	2776	74303	26.77
5	Germany	1926	27738	14.40
6	Sweden	1202	25620	21.31
7	Netherlands	846	35396	41.84
8	Italy	792	20024	25.28
9	Japan	740	10567	14.28
10	France	642	17502	27.26
11	Brazil	640	7991	12.49
12	Spain	638	13056	20.46
13	China	481	6389	13.28
14	India	461	7308	15.85
15	Switzerland	430	9042	21.03
16	Denmark	415	12326	29.70
17	Israel	377	5869	15.57
18	South Korea	369	5647	15.30
19	South Africa	356	7466	20.97
20	Norway	340	7569	22.26

The top five organisations publishing OT publications in the last 20-years are the department of occupational science and occupational therapy, the University of Toronto, Toronto, Canada, department of health sciences, Lund University, Lund, Sweden, school of rehabilitation science, McMaster University, Hamilton, Canada, school of health and rehabilitation sciences, the University of Queensland, Brisbane, Australia and department of occupational therapy, Colorado state university, Fort Collins, USA ( Table 3). Among the Top-20 university of British Columbia, Vancouver, Canada, received the highest citations (cpd = 200.50).

**Table 3.** Top-20 occupational therapy organisations.

Organization	Country	documents	citations	CPD
Department of occupational science and occupational therapy, university of Toronto, Toronto	Canada	41	407	9.93
Department of health sciences, Lund university, Lund,	Sweden	36	624	17.33
School of rehabilitation science, McMaster university, Hamilton, on,	Canada	36	614	17.06
School of health and rehabilitation sciences, the university of Queensland, Brisbane,	Australia	35	468	13.37
Department of occupational therapy, Colorado state university, fort Collins, co,	United States	30	630	21.00
Department of occupational therapy, university of Illinois at Chicago	United States	29	318	10.97
Department of occupational therapy, school of rehabilitation sciences, Iran university of medical sciences, Tehran,	Iran	27	68	2.52
National research centre for the working environment, Copenhagen	Denmark	26	632	24.31
Program in occupational therapy, Washington university school of medicine, St. Louis, Mo,	United States	26	355	13.65
Harvard medical school, Boston, ma,	United States	25	2723	108.92
Faculty of health sciences, university of Sydney, Sydney, NSW,	Australia	24	1238	51.58
School of occupational therapy, college of medicine, national Taiwan university, Taipei	Taiwan	24	359	14.96
Division of occupational therapy, school of health and rehabilitation sciences, university of Queensland, Brisbane	Australia	22	576	26.18
McMaster university, Hamilton	Canada	22	3172	144.18
University of British Columbia, Vancouver	Canada	22	4411	200.50
American occupational therapy association, Bethesda, md,	United States	21	227	10.81
Department of physical therapy, university of Toronto, Toronto	Canada	21	184	8.76
School of occupational therapy and social work, Curtin university, Perth, WA	Australia	21	142	6.76
School of physical and occupational therapy, McGill university, Montreal.	Canada	21	281	13.38
Division of occupational therapy, school of health and rehabilitation sciences, university of Queensland, Brisbane	Australia	20	399	19.95

Among the top researcher, Brown T (n=128) was identified as a leading author with the maximum number of articles in the field of OT, followed by Eklund M (n=88), Kottorp A (n= 41), Rodger S (n=88), Ziviani J. (n=76) and Mackenzie I. (n=67). The top authors' citation per document and lifetime h Indexed are provided in Table 4. Further, the top-20 highly cited publications are listed in Table 5. The article published in 2017, received the 2309 citation was the highly cited article in the lancet. We observed a negative correlation (-0.14278) when comparing citations with the total year after publication.

**Table 4.** Top-authors in occupational therapy research.

Sl no	Author	Scopus id	Affiliation	Country	Doc	citations	cpd	h index
1	Brown T.	35228602300	Monash University, Melbourne.	Australia	128	1137	8.88	27
2	Eklund M.	56216675200	Institutionen för Hälsovetenskaper, Lund.	Sweden	88	1742	19.80	37
3	Rodger S.	7005081814	The University of Queensland, Brisbane.	Australia	88	2048	23.27	35
4	Ziviani J.	6603664282	The University of Queensland, Brisbane.	Australia	76	2081	27.38	43
5	Mackenzie L.	7006703790	The University of Sydney, Sydney	Australia	67	871	13.00	21
6	Clemson L.	6602125528	The University of Sydney School of Health Sciences, Sydney	Australia	63	1453	23.06	37
7	Fleming J.	7401457123	The University of Queensland, Brisbane.	Australia	60	1207	20.12	35
8	Law M.	7202653007	McMaster University, Hamilton	Canada	58	2623	45.22	63
9	Gitlin L.N.	7003860203	Johns Hopkins University, Baltimore.	United States	53	3048	57.51	50
10	Gustafsson L.	7203014989	Griffith University, Brisbane	Australia	53	476	8.98	16
11	Kottorp A.	6506079508	Malmö Högskola, Malmo	Sweden	52	751	14.44	26
12	Mckenna K.	7102801655	The University of Queensland, Brisbane	Australia	51	1182	23.18	32
13	Abo M.	7004758531	The Jikei University School of Medicine, Tokyo	Japan	50	584	11.68	27
14	Kielhofner G.	7007094254	University of Illinois at Chicago, Chicago	United States	49	1166	23.80	33
15	Gutman S.A.	7004894255	Columbia University, New York	United States	48	382	7.96	15
16	Lannin N.A.	6602527646	Affiliation information is based on the most recent publication Monash University, Melbourne	Australia	48	753	15.69	34
17	Mccluskey A.	55523738500	The University of Sydney School of Health Sciences, Sydney	Australia	48	1171	24.40	22
18	Iwarsson S.	26643085000	Institutionen för Hälsovetenskaper, Lund.	Sweden	46	1599	34.76	43
19	Tham K.	7004310672	Malmö Högskola, Malmo	Sweden	44	1078	24.50	32
20	Lloyd C.	7202193315	Griffith University, Brisbane,	Australia	43	818	19.02	24

**Table 5.** Top-20 highly cited OT publications.

Sl No	Article	Citations	Age of publications
1	Livingston, G., Sommerlad, A., Orgeta, V., Costafreda, S. G., Huntley, J., Ames, D., … Mukadam, N. (2017). Dementia prevention, intervention, and care. The Lancet, 390(10113), 2673-2734. doi:10.1016/S0140-6736(17)31363-6	2309	3
2	Shepard, C. W., Finelli, L., & Alter, M. J. (2005). Global epidemiology of hepatitis C virus infection. Lancet Infectious Diseases, 5(9), 558-567. doi:10.1016/S1473-3099(05)70216-4	2199	15
3	Bateman, E. D., Hurd, S. S., Barnes, P. J., Bousquet, J., Drazen, J. M., FitzGeralde, M., … Zar, H. J. (2008). Global strategy for asthma management and prevention: GINA executive summary. European Respiratory Journal, 31(1), 143-178. doi:10.1183/09031936.00138707	2195	12
4	Ringleb, P. A., Bousser, M. -., Ford, G., Bath, P., Brainin, M., Caso, V., … Wardlaw, J. (2008). Guidelines for management of ischaemic stroke and transient ischaemic attack 2008. Cerebrovascular Diseases, 25(5), 457-507. doi:10.1159/000131083	2076	12
5	Schweickert, W. D., Pohlman, M. C., Pohlman, A. S., Nigos, C., Pawlik, A. J., Esbrook, C. L., … Kress, J. P. (2009). Early physical and occupational therapy in mechanically ventilated, critically ill patients: A randomised controlled trial. The Lancet, 373(9678), 1874-1882. doi:10.1016/S0140-6736(09)60658-9	1856	11
6	Langhorne, P., Bernhardt, J., & Kwakkel, G. (2011). Stroke rehabilitation. The Lancet, 377(9778), 1693-1702. doi:10.1016/S0140-6736(11)60325-5	1255	9
7	Gillespie, L. D., Robertson, M. C., Gillespie, W. J., Sherrington, C., Gates, S., Clemson, L. M., & Lamb, S. E. (2012). Interventions for preventing falls in older people living in the community. Cochrane Database of Systematic Reviews, 2012(9) doi:10.1002/14651858.CD007146.pub3	1216	8
8	Baumeister, R. F., Campbell, J. D., Krueger, J. I., & Vohs, K. D. (2003). Does high self-esteem cause better performance, interpersonal success, happiness, or healthier lifestyles? Psychological Science in the Public Interest, 4(1), 1-44. doi:10.1111/1529-1006.01431	1041	17
9	Flora, G., Gupta, D., & Tiwari, A. (2012). Toxicity of lead: A review with recent updates. Interdisciplinary Toxicology, 5(2), 47-58. doi:10.2478/v10102-012-0009-2	1024	8
10	Amini, D. A., Kannenberg, K., Bodison, S., Chang, P. -., Colaianni, D., Goodrich, B., … Lieberman, D. (2014). Occupational therapy practice framework: Domain & process 3rd edition. American Journal of Occupational Therapy, 68, S1-S48. doi:10.5014/ajot.2014.682006	1007	6
11	Alter, M. J. (2007). Epidemiology of hepatitis C virus infection. World Journal of Gastroenterology, 13(17), 2436-2441. doi:10.3748/wjg.v13.i17.2436	969	13
12	Sambrook, P., & Cooper, C. (2006). Osteoporosis. Lancet, 367(9527), 2010-2018. doi:10.1016/S0140-6736(06)68891-0	934	14
13	Winstein, C. J., Stein, J., Arena, R., Bates, B., Cherney, L. R., Cramer, S. C., … Zorowitz, R. D. (2016). Guidelines for adult stroke rehabilitation and recovery: A guideline for healthcare professionals from the american heart Association/American stroke association. Stroke, 47(6), e98-e169. doi:10.1161/STR.0000000000000098	918	4
14	Ohgaki, H., & Kleihues, P. (2005). Epidemiology and etiology of gliomas. Acta Neuropathologica, 109(1), 93-108. doi:10.1007/s00401-005-0991-y	902	15
15	Shi, H., Magaye, R., Castranova, V., & Zhao, J. (2013). Titanium dioxide nanoparticles: A review of current toxicological data. Particle and Fibre Toxicology, 10(1) doi:10.1186/1743-8977-10-15	862	7
16	Baumeister, R. F., Campbell, J. D., Krueger, J. I. I., & Vohs, K. D. (2003). Does high self-esteem cause better performance, interpersonal success, happiness, or healthier lifestyles? Psychological Science in the Public Interest, Supplement, 4(1) doi:10.1111/1529-1006.01431	828	17
17	Litz, B. T., Stein, N., Delaney, E., Lebowitz, L., Nash, W. P., Silva, C., & Maguen, S. (2009). Moral injury and moral repair in war veterans: A preliminary model and intervention strategy. Clinical Psychology Review, 29(8), 695-706. doi:10.1016/j.cpr.2009.07.003	818	11
18	Van Tulder, M., Becker, A., Bekkering, T., Breen, A., Del Real, M. T. G., Hutchinson, A., … Malmivaara, A. (2006). Chapter 3: European guidelines for the management of acute nonspecific low back pain in primary care. European Spine Journal, 15(SUPPL. 2), S169-S191. doi:10.1007/s00586-006-1071-2	817	14
19	Wallace, D. V., Dykewicz, M. S., Bernstein, D. I., Blessing-Moore, J., Cox, L., Khan, D. A., … Tilles, S. A. (2008). The diagnosis and management of rhinitis: An updated practice parameter. Journal of Allergy and Clinical Immunology, 122(2 SUPPL.), S1-S84. doi:10.1016/j.jaci.2008.06.003	808	12
20	Roley, S. S., DeLany, J. V., Barrows, C. J., Brownrigg, S., Honaker, D., Sava, D. I., … Youngstrom, M. J. (2008). Occupational therapy practice framework: Domain & process 2nd edition. American Journal of Occupational Therapy, 62(6), 625-683. doi:10.5014/ajot.62.6.625	795	12

**Figure 4.**
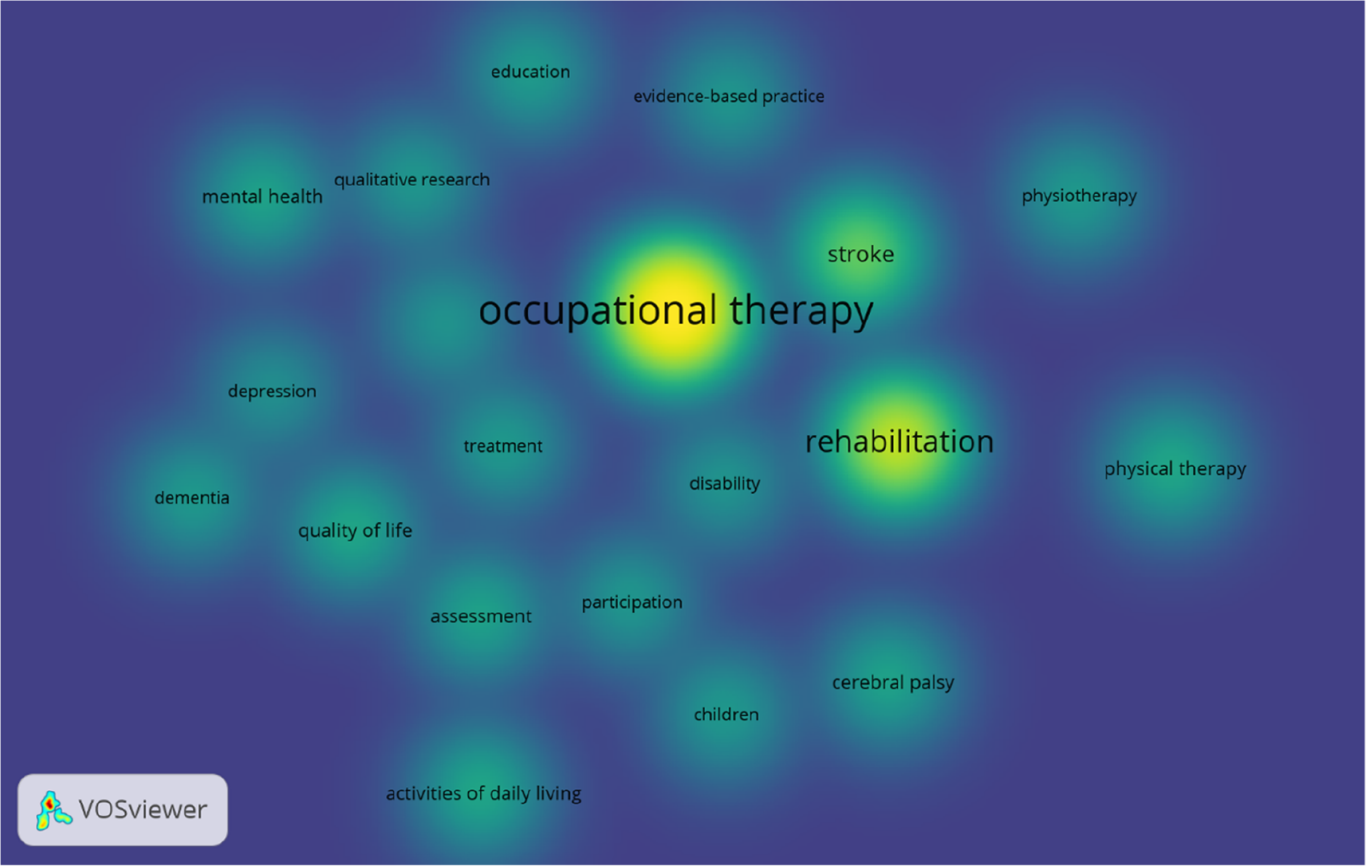
Density visualisation of keywords in occupational therapy publications.

Our study enlisted 20 author keywords commonly used in OT literature. Overall, the best 20 keywords were identified, with at least 50 occurrences in the OT publications ( Figure 4).

## Discussion

Over the last twenty years, overall OT publication output steadily increased, indicating the profession's growth in terms of research, and its dissemination is not significantly influencing the modern era of digitalization. Publication in non-OT-specific journals is three times more compared to OT-specific journals. A similar trend was also observed in recent studies ( T. Brown *et al.,* 2019; Gutman *et al.,* 2017; Folha *et al.,* 2017; T. Brown, Ho *et al.,* 2018; T. Brown, Gutman, and Ho 2018; Folha *et al.,* 2019). This increase may reflect the growth of research activities in OT across the globe ( Folha *et al*., 2019). Occupational therapy works along with medical and other allied health professionals. It allows occupational therapies to work as a part of the multidisciplinary team. Similarly, it opens the door to interdisciplinary research opportunities. That multidisciplinary research is used to publish in either medical-related or multidisciplinary journals. Those journals have high JIF compared to OT journals ( T. Brown *et al*., 2019; Gutman *et al.*, 2017; Folha *et al.*, 2017; T. Brown, Ho *et al.*, 2018; T. Brown, Gutman, and Ho 2018; Folha *et al.*, 2019). A recent study found that non-OT-specific journals have three times more JIF than OT-specific journals ( Folha *et al.*, 2019). These multidisciplinary and medical-oriented journals have more readers than OT-specific journals, giving more visibility and increasing the chance of receiving more citations. It may also encourage OT researchers to publish more in non-OT-specific journals ( T. Brown *et al*., 2019; Gutman *et al.*, 2017; Folha *et al.*, 2017; T. Brown, Ho *et al*., 2018; T. Brown, Gutman, and Ho 2018; Folha *et al*., 2019).

A total of five journals, such as AJOT, BJOT, AOTJ, SJOT, and CJOT, published half of the OT-specific journals articles. Previous studies also made similar observations ( T. Brown and Gutman 2019; T. Brown, Gutman, and Ho, 2018). These journals are well-known among the OT profession and have a long publishing history ( T. Brown and Gutman, 2019). AJOT, BJOT, AOTJ, and CJOT were published by prominent national organizations of OT and recognised by the global OT community ( T. Brown and Gutman, 2019). These journals' publication frequencies are more and published many articles per issue compared to other journals. Among these 16 OT journals, two journals, BrJOT and IrJOT, are new in the Scopus database and have data for the past three years. Hence, those journals will have a lower number of articles. According to the previous study, Scopus indexed OT-specific journal numbers was 14 ( T. Brown and Gutman, 2019). Among these two new journals, BrJOT published many articles in three years and placed under the best 20 journals that published OT-specific articles. OT-specific journals published one-third of total OT publications. Several OT-specific journals are published globally and have an online publication ( Cruz *et al.,* 2019) but are not included in the Scopus database. Including all those OT-specific Journals might change the scenario of citation matrices of occupational therapy journals because occupational therapy journals tend to get more citations in for OT-specific journals.

Out of 20 top journals that produced the maximum articles in occupational therapy, the first eight are OT-specific journals. There is a normal phenomenon because these journals published OT-specific articles. ErgoR received more minor citations among those ten OT-specific journals due to its publication language. This is the only journal listed in the top twenty published in Germany. All other periodicals are published in the English language. AJOT, BJOT, AOTJ, SJOT, and CJOT received more citations in terms of overall citation. It may be due to their large volume of publications ( T. Brown and Gutman, 2019; T. Brown, Gutman, Ho, *et al.*, 2018). AJOT, BJOT, AOTJ, SJOT, and CJOT received more than ten citations per document. *Archives of Physical Medicine and Rehabilitation* received more citations per document than any other journal listed in top-20, and it has the highest CiteScore and JIF. Among the OT-specific journals, POTP had the highest CiteScore (2.9), and AJOT had the highest JIF (2.246).

The US, Australia, UK, Canada, Germany, Sweden, Netherlands, and Italy are the top most country in terms of numbers of published articles, and this is different from that of the previous study ( T. Brown and Gutman 2019; T. Brown, Gutman, Ho, *et al.,* 2018). The US published approximately one-third of total OT publications (n=9517). Other study findings also revealed that the US is the top OT article ( T. Brown, Gutman, Ho, *et al.,* 2018). The US received many citations (216810) for the published documents. US is the country where OT originated from and also have a good education and research system in OT, which may be the reason for the huge number of the publication. The US is also the host country for a large number of OT-specific and non-OT-specific journals. This could also be a reason for the huge number of publications in occupational therapy. But citation per document is more minor for US articles than the Netherlands, Denmark, Italy, France, and Norway, with very few publications compared to the US. It may be due to the lack of availability of OT-specific journals in their geographic location, which triggered them to publish their research in non-OT specific journals with higher quality than OT-specific journals.

We found that the US, Australia, UK, and Canada are the leading countries in international collaborations in OT publications. The size of the level and circle indicate the weightage of collaborative activity. A bigger size represents more collaborations, and smaller size means fewer collaborations. The line between two nodes and their densities represents their link and strength ( Figure 3). Colour expressed different clusters levels based on international collaborations ( Van Eck and Waltman, 2019).

Among the top-20 organizations, 18 are either universities or entities of a university. This happens because universities are mainly placed in the urban set up with all necessary support such as research culture among academicians, well-equipped libraries with academic resources, affiliated hospitals, client participation, internal funding, grant office supports ( T. Brown *et al.,* 2019). All this is favourable for more research output, which may cause university domination's top twenty organization list. Our study found only one organization in this top list: research institutes from Denmark and one occupational therapy association of USA. Though the US alone produced many OT articles, only five organizations are listed in the top 20, compared to US Canada (n=6) and Australia (n=5). Despite having a high volume of publications, less representation of US organization in the top list was also observed in previous studies ( T. Brown, Gutman, Ho, *et al.*, 2018; Man *et al.*, 2019). Other countries that are listed in top organizations are Denmark (n=1), Iran (n=12), Sweden (n=1), and Taiwan (n=1).

Australia (n=11) has the highest representation in the top author list, followed by Canada (n=1) Sweden (n=4), US (n =3), and Japan (n=1). The US authors are less in the top list instate having high publication volume. UK authors produce more publications after US authors, but there are no authors from the UK listed in the top 20 author list. Law M. from McMaster University, Hamilton, Canada had the highest h indexed (63) and Gitlin L. N from Johns Hopkins University, Baltimore, united states received the highest citation per document (57.51) among top-20 authors. Our study found Brown T as a top author in publication number. The finding of T. Brown, Gutman, Ho, *et al.* (2018) supported our study.

Out of the top-twenty highly cited articles for 2001 to 2020, four papers were published in 2008, two each in 2005, 2006, 2009, and 2012, one each in 2002, 2003, 2004, 2007, 2013, 2014, 2016, and 2017. Our study does not find any relation with year after with more citations. Our findings opposed previous studies' findings, which observe a long citation window helps an article gather maximum citation ( Gutman *et al.,* 2017; T. Brown *et al.,* 2019; T. Brown, Gutman, Ho, *et al.*, 2018; T. Brown *et al.,* 2017). Most of the highly cited papers are published in non-OT-specific journals. Most of the highly cited studies published non-OT-specific journals like earlier studies. Those non-OT journals are generally high-quality medical journals ( Gutman *et al.,* 2017; T. Brown, Gutman, Ho, *et al.*, 2018; T. Brown *et al.*, 2019; T. Brown *et al.*, 2017; T. Brown, Ho *et al.*, 2018). It may happen due to the increased pressure to publish in journals with good impact factors or CiteScore. The quality of journals provides more visibility, which helps the author get more readership and citation because it helps in a grant application and promotion ( Gutman *et al.* 2017; T. Brown *et al.*, 2019).

VOSviewer density visualisation ( Figure 4), by default, uses blue, green, and yellow to represent the density visualisation ( Van Eck and Waltman, 2019). The yellow colour indicates the number of items in the neighbourhood of a point and the higher weights of the neighbouring items. Out of the total best 20 keywords, “occupational therapy”, “rehabilitation,” and “stroke”, “physical therapy” and “activities of daily living” are five common keywords used more frequently in OT research, which are in yellow ( Figure 4). Occupational therapy and rehabilitation are commonly used as keywords because these two words are identical terms for the occupational therapy profession. The authors used these two words to make their research visible in the electronic search. Stoke is most probably the oldest and strongest research field, where occupational therapist works across the globe. Physical therapy is a common allied health profession occupational therapists may collaborate and publish together. Activities of daily living are one of the core practice areas of the occupational therapist.

## Conclusion

This study retrieved scientometric information from the Scopus database and listed 25 journals along with their CiteScore and Journal Impact Factor. It was observed OT articles were published three times more in non-OT-specific journals, indicating that OT research is significantly overlapping with other disciplines of medicine. Hence, the investigation should not conclude a literature search with only OT-specific journals. The SJOT is one of the core OT journals but listed under subject category of “public health, environmental and occupational health” rather than the category of “occupational therapy”.

### Key points

1) From Scopus database, twenty Journals were identified, which published a maximum number of occupational therapy articles.
2) Scopus database indexed a total of 16 OT-specific journals. Scopus database also included two OT practice magazines in their database.
3) VOSviewer software is an open-access tool that can be used as a cost-effective method for any scientometric analysis.
4) BJOT, AJOT, AOTJ, SJOT, and CJOT are the leading occupational therapy journals in the number of published items.

## Data availability

### Underlying data

Mendeley Data: Scopus Based Occupational Therapy Research (2001-2020). https://doi.org/10.17632/yp7xjg4zs3.2 (Sau and Nayak, 2022)

This project contains the following underlying data:

Data File “20 best source which published maximum number of article” contains analysis of the data obtained from Scopus for 2001-2020. The analysis includes 20 best sources, 20 best cited documents, 20 best authors, 20 best countries, 20 best organisations, 20 highest used keywords, year-wise publication analysis, and OT-specific journals details.

Data files “Scopus-935-Analyze-Source”, “Scopus-981-Analyze-Source”, “Scopus-984-Analyze-Source”, “Scopus-1029-Analyze-Source”, “Scopus-1038-Analyze-Source”, “Scopus-1186-Analyze-Source”, “Scopus-1365-Analyze-Source”, “Scopus-1287-Analyze-Source”, “Scopus-1366-Analyze-Source”, “Scopus-1400-Analyze-Source”, “Scopus-1508-Analyze-Source”, “Scopus-1657-Analyze-Source”, “Scopus-1784-Analyze-Source”, “Scopus-1785-Analyze-Source”, “Scopus-1819-Analyze-Source”, “Scopus-1776-Analyze-Source”, “Scopus-1868-Analyze-Source”, “Scopus-1962-Analyze-Source”, “Scopus-2234-Analyze-Source”, “Scopus-2479-Analyze-Source” respectively contains the data obtained for the years 2001, 2002, 2003, 2004, 2005, 2006, 2007, 2008, 2009, 2010, 2011, 2012, 2013, 2014, 2015, 2016, 2017, 2018, 2019 and 2020 respectively with the keywords for search.

Data are available under the terms of the Creative Commons Attribution 4.0 International license (CC-BY 4.0).
