# Supplementary material for: Scopus based bibliometric and scientometric analysis of occupational therapy publications from 2001 to 2020
Source: F1000Res. 2023 Jun 22;11:155. Originally published 2022 Feb 8. [Version 3] doi: 10.12688/f1000research.108772.3 (PMC11959257; doi:10.12688/f1000research.108772.3)
Supplement: Peer review history

# Reviewer report: Reviewer response for version 3

**DOI:** 10.5256/f1000research.151640.r371721

**Published:** 2025-03-31

**Authors:** Kim Walder

**Affiliations:** 1 Griffith University, Queensland, Australia

Thank you for the opportunity to review this manuscript. 

 As per previous feedback, the rigour of this analysis is impacted by limiting the search to one database. Although the authors provide rationale for this decision, it also runs the risk of missing publications.

 Unfortunately grammar and syntax makes it challenging to follow in places. 

 Unfortunately, this study is now quite dated as there have potentially been marked changes since 2020 in publishing of occupational therapy literature, with a significant increase in advocating for research coming out of global majority countries, and non-Western- lens which has also resulted in changes to where papers may be published. In other words, the currency of findings is questionable. 

 Several occupational therapy specific journals have been omitted from this study, potentially due to limiting the search to Scopus. 

 These include (but are not limited to)

 - Chilean Journal of Occupational Therapy/Revista Chilena de Terapia Ocupacional

 - Journal of Acute Care for Occupational Therapy

 -Asian Journal of Occupational Therapy

 - Journal of Occupational Therapy Education

 - South African Journal of Occupational Therapy

 - Columbian Occupational Therapy Journal/ Revista Ocupación Humana 

 - French Journal of Occupational Therapy

 The authors, themselves, identify the significant limitation caused by omitting journals not within the SCOPUS database. 

 Occupational science as a field of research and also focus of publications has not been mentioned at all, yet warrants discussion given the overlap with occupational therapy research and publishing. 

 Once sentence has Australia listed twice. Is there another country meant to be listed as having "solid" international collaboration (Figure 3). Can you please specify what you define as "solid".

 Capitalisation for proper nouns needs attention.

 The author listed as one of the leading authors is L. Mackenzie, not I. Mackenzie.

 How did you determine "best" keywords. Quantity does not mean quality. 

 The point that occupational therapy is rapidly moving into non-medical areas of practice is not mentioned in the discussion. In fact, the paper is written from a rather biomedical view of occupational therapy e.g. in the description of OTs working alongside medical and other allied health disciplines. This not a very contemporary nor global perspective of occupational therapy. 

 Correct me if I am not familiar with your referencing style, but in-text citations do not appear to consistency follow a particular referencing style. 

 The authors incorrectly state that countries such as Netherlands, Denmark, Italy, France, and Norway lack OT specific journals - yet there is the Scandinavian Journal of Occupational Therapy which would be geographically relevant to Denmark and Norway and the French Journal of Occupational Therapy. Further, the relevance of geography is not that clear and this argument should be elaborated on further. 

 I acknowledge the argument re LMIC countries and whilst this may be true, findings are flawed due to the exclusion of some journals from LMIC countries. I n fact, limiting the search to the term "occupational" leads to cultural and linguistic biases.

 There is inclusion of new findings in discussion section. These sections needs to be explicitly delineated. 

 The manuscript could be significantly enhanced by providing a more critical discussion with implication for the profession, its evidence base, professional identity, recognition and status.

 I am assuming the term "stoke" is meant to be "stroke". The final three sentences of the discussion seem out of place and their intent is not clear. In fact it waters down the discussion even more in my opinion. For example is the mention of collaborations with physical therapists a finding or a thought thrown in by the authors. The same question is asked of "activities of daily living".

 The conclusion warrants careful revision based on the methodological flaws which limit the credibility of the conclusions drawn. I am not clear what the authors are referring to as "knowledge translations" in the second sentence in the conclusion. This seems to be different from the common understanding and conceptualisation of knowledge translation. Perhaps elaboration will help the authors convey their intended meaning. 

 Discussion in relation to implications for the profession  and future research could be greatly enhanced, especially if the discussion was reworked from using a more critical lens.

Is the work clearly and accurately presented and does it cite the current literature?

Partly

If applicable, is the statistical analysis and its interpretation appropriate?

Partly

Are all the source data underlying the results available to ensure full reproducibility?

No

Is the study design appropriate and is the work technically sound?

No

Are the conclusions drawn adequately supported by the results?

No

Are sufficient details of methods and analysis provided to allow replication by others?

Partly

Reviewer Expertise:

Occupational therapy professional identity; literature reviews; qualitative research; occupational science; occupational adaptation;

I confirm that I have read this submission and believe that I have an appropriate level of expertise to confirm that it is of an acceptable scientific standard, however I have significant reservations, as outlined above.

# Reviewer report: Reviewer response for version 3

**DOI:** 10.5256/f1000research.151640.r330465

**Published:** 2024-10-23

**Authors:** Miguel Valencia-Contrera

**Affiliations:** 1 Faculty of Nursing, Universidad Andres Bello, Santiago, Santiago Metropolitan Region, Chile

The authors address a phenomenon that is both relevant and necessary to study, particularly in the field of occupational therapy. They introduce the phenomenon in a comprehensive manner, providing context, highlighting its relevance, outlining the state of the art, and identifying the knowledge gap they aim to address. However, I believe some aspects require further elaboration or explanation.

1. The first point I consider important is the discussion of the most influential journals in the field. While the authors mention the five leading journals (which account for more than half of the publications in occupational therapy-specific journals), I believe it is necessary to conduct an analysis based on Bradford’s Law. This law suggests that the majority of articles on a specific topic are published in a small number of journals, forming a core (first zone), followed by a larger set of journals (second zone), which contain fewer articles on the topic, and finally a third zone follows the same logic. If the authors disagree with my suggestion, I consider it important to explain why Bradford’s Law was not applied in the analysis of their data.
2. The second point I find important is the impact of the COVID-19 pandemic. Was the productivity observed in 2020 influenced by the pandemic? Is the density of the keywords affected by articles published on the pandemic? What does the current state of the art indicate in this regard? I believe it is necessary to analyze this aspect, at least in a general manner.

Congratulations to the authors on this article, which will undoubtedly contribute to the state of the art in this field. I hope my comments will help improve the quality of the study.

 Sincerely, The reviewer.

Is the work clearly and accurately presented and does it cite the current literature?

Yes

If applicable, is the statistical analysis and its interpretation appropriate?

Not applicable

Are all the source data underlying the results available to ensure full reproducibility?

Yes

Is the study design appropriate and is the work technically sound?

Yes

Are the conclusions drawn adequately supported by the results?

Partly

Are sufficient details of methods and analysis provided to allow replication by others?

Partly

Reviewer Expertise:

Specialist in management and editing of scientific journals. Methodologist in scientific literature reviews.

I confirm that I have read this submission and believe that I have an appropriate level of expertise to confirm that it is of an acceptable scientific standard, however I have significant reservations, as outlined above.

# Reviewer report: Reviewer response for version 2

**DOI:** 10.5256/f1000research.146052.r173017

**Published:** 2023-05-25

**Authors:** Sureshkumar Kamalakannan

**Affiliations:** 1 Department of Social Work, Education and Community Well-being, Northumbria University, Newcastle-upon-Tyne, UK; 2 Public Health Foundation of India (PHFI), South Asia Centre for Disability Inclusive Development and Research (SACDIR), Indian Institute of Public Health - Hyderabad (IIPH-H), Hyderabad, Telangana, India

It is good to see that the authors have revised the manuscript based on the reviewer's comments. Please proofread the manuscript. For example Low and Middle-Income Country has an acronym LIMC.

 I approve this submission.

 Best Wishes

 Suresh

Is the work clearly and accurately presented and does it cite the current literature?

Yes

If applicable, is the statistical analysis and its interpretation appropriate?

Yes

Are all the source data underlying the results available to ensure full reproducibility?

Yes

Is the study design appropriate and is the work technically sound?

Yes

Are the conclusions drawn adequately supported by the results?

No

Are sufficient details of methods and analysis provided to allow replication by others?

Partly

Reviewer Expertise:

NA

I confirm that I have read this submission and believe that I have an appropriate level of expertise to confirm that it is of an acceptable scientific standard.

# Response

**Published:** 2023-06-15

**Authors:** Yogendra Nayak

**Affiliations:** Manipal Academy of Higher Education, India

Thank you for this. We have corrected the error and revised the manuscript.

# Reviewer report: Reviewer response for version 1

**DOI:** 10.5256/f1000research.120194.r140811

**Published:** 2022-07-20

**Authors:** Sureshkumar Kamalakannan

**Affiliations:** 1 Department of Social Work, Education and Community Well-being, Northumbria University, Newcastle-upon-Tyne, UK; 2 Public Health Foundation of India (PHFI), South Asia Centre for Disability Inclusive Development and Research (SACDIR), Indian Institute of Public Health - Hyderabad (IIPH-H), Hyderabad, Telangana, India

This is an important work implying the need for OT (occupational therapy) research development. The primary concern is related to the selection of only one database for this research study and the rationale/importance of this work in the current context. This needs to be justified and details related to the rationale must be added. The other concern is related to the discussion conclusion sections - where the authors highlight several aspects related to the results from this research but fail to connect the findings with implications for OT research, professional development, policies, and practice. Particularly the implications for LMICs (Low and Middle Income Countries). This study highlights OT research developing in 5 high-income nations and misses to include what could be done in the rest of the world to bring OT research to the standards found in those 5 high-income nations. The conclusions are also not directly relevant. These issues require revision. Additionally, the language requires proofreading and revision.

 I congratulate the authors for their efforts and suggestions to revise the manuscript based on my comments.

Is the work clearly and accurately presented and does it cite the current literature?

Yes

If applicable, is the statistical analysis and its interpretation appropriate?

Yes

Are all the source data underlying the results available to ensure full reproducibility?

Yes

Is the study design appropriate and is the work technically sound?

Yes

Are the conclusions drawn adequately supported by the results?

No

Are sufficient details of methods and analysis provided to allow replication by others?

Partly

Reviewer Expertise:

Disability, Public Health and Epidemiology

I confirm that I have read this submission and believe that I have an appropriate level of expertise to confirm that it is of an acceptable scientific standard, however I have significant reservations, as outlined above.

# Response

**Published:** 2023-03-23

**Authors:** Yogendra Nayak

**Affiliations:** Manipal Academy of Higher Education, India

Authors are thankful for the reviewer comment. We are writing the following responses with the revision of the manuscript.

1. We have justified why we used one databased for our study in methodology sections under the subheadings “source of data”. Citations vary between databases and it is based on the number of journals indexed in that database. We selected Scopus for this study because it includes more OT-specific journals compared to WOS and overall indexed more journals compared to WOS. The citation count was calculated within the database. Due to extensive coverage, Scopus has a more citable item and a probability of getting more citations to count.
2. The suggestions of LIMC is included in the discussion sections of revised manuscript.
3. Conclusion is modified according to suggestions, incorporation of the implications for further research in the field of occupational therapy. 
4. Language proofing taken care and the necessary changes are made in the revised manuscript.
